# Association Between Machine Translation Post-Editing and Post-Editors’ Cognition: A Three-Level Meta-Analysis Based on Eye-Tracking Evidence

**DOI:** 10.3390/bs16030365

**Published:** 2026-03-04

**Authors:** Feng Wang, Hong Xie, Xiang Zhang

**Affiliations:** 1 School of Translation Studies, Shandong University, Weihai 264209, China; 2 Faculty of Languages and Translation, Macao Polytechnic University, Macao, China; zhangxiang@mpu.edu.mo

**Keywords:** machine translation, cognition, meta-analysis, eye-tracking, priming effect

## Abstract

Prior research has overlooked interdependent effect sizes and moderating factors between machine translation post-editing (MTPE) and the post-editor’s cognition. To fill this void, the study employs a three-level meta-analysis. A total of 19 high-quality studies, with an average quality score of 8.158, were included in the analysis. These included studies encompass 193 effect sizes and 492 participants. The findings indicate a positive link between MTPE and the post-editor’s cognition (r = 0.474), suggesting higher levels of cognitive engagement in post-editing tasks. Among the moderators examined, several study-level characteristics were associated with variability in effect sizes. These characteristics include whether PE attitudes were reported, text types, cognition measurement tools, and cognitive indicators. In contrast, studies that reported MTPE attitudes showed a different pattern of effect sizes. Overall, this study provides rigorous evidence on the multifaceted impacts of MTPE on translators’ cognition. It also clarifies how dependent effect sizes should be modeled in translation cognition research.

## 1. Introduction

A cogent statement by Wagner (1985) suggests that post-editing (PE) entails correcting a pre-translated text instead of undertaking translation from scratch. In other words, the task of a post-editor is to edit, modify and correct a pre-translated text that has been processed by machine translation (MT) systems (Harold, 2003). Recently, there has been a surge of interest in PE within the user community due to the increasing quality of MT outputs and the availability of free, high-quality software for PE (Balling et al., 2014). The accessibility of a wide array of translation data derived from MT, which serves as the basis for studying PE, further propels this research agenda. These research interests always cover translation quality assessment (Mitchell et al., 2014; Ortiz-Boix & Matamala, 2017; Hudecova et al., 2024), error types and correction (Bassil & Alwani, 2012; Daems et al., 2017a; Vardaro et al., 2019), as well as allocation of attentional resources and the cognitive effort of post-editors (Lacruz et al., 2014; Lacruz & Shreve, 2014; Sánchez-Gijón et al., 2019; Qian et al., 2022).

Furthermore, the integration of PE into digital translation workflows raises a question that has not been addressed in traditional human translation (HT) or its comparison with MT (Balling et al., 2014). The comparisons between MT and HT, along with the contests among diverse MT systems, spur the optimization of MT. This optimization further improves the algorithms used in neural machine translation (NMT), automatic machine translation (AMT), and hybrid machine translation (HMT). Notwithstanding the progress in translation productivity, MT outputs still exhibit deficiencies in handling logical structures, semantic subtleties, and contextual factors (Moorkens et al., 2018). These deficiencies often lead to “an unfinished text” (Harold, 2003) or a text devoid of context. Such problems are typically remedied through post-editing. In this sense, the post-editing results might be influenced by two factors: first, the post-editor’s acceptance of the MT output; second, the post-editor’s preferences and his/her cognition invested in modifying the MT output.

In this context, the post-editor’s cognition encompasses a vast array of activities and processes related to the monitoring, understanding, retention, retrieval, and restructuring of MT outputs. This sequence of mental operations may occur either consciously or subconsciously, as noted by Bayne et al. (2019). In the context of MTPE, such cognition pertains to the nature and extent of the cognitive processes in which the post-editor must engage. These processes are activated to rectify specific inadequacies in pre-translated texts, as posited by Krings (2001). Regrettably, the cognitive process, by its very nature, eludes direct observation because of the limitations of prior neuro-cognitive technology. In recent years, with the emergence of non-invasive eye-tracking instruments, such as Eyelink 1000 plus and Tobii T120, these limitations have been gradually alleviated. Therefore, to more comprehensively penetrate the intricate realm of the post-editor’s cognition, certain scholars have developed a substantial interest in its association with MTPE. Among the extant studies, the cognitive effort, load and memory of editors are perceived as metrics that reflect the quality and efficiency of MT (e.g., Lacruz et al., 2014; Lacruz & Shreve, 2014; Sánchez-Gijón et al., 2019; X. Wang et al., 2024).

From a more nuanced perspective of the previous literature, pauses (O’Brien, 2006), editing time (Koponen et al., 2012), and the word-based human edit rate (Huang & Carl, 2022) are commonly used metrics to reflect the cognitive effort, load, and memory of editors. Researchers have employed these metrics to examine the link between MTPE and editors’ cognition, but the reported effect sizes varied considerably across studies, ranging from essentially 0 to 0.487. This wide range reflects substantial variation in the observed outcomes and highlights the need for a meta-analytic approach to estimate the overall average effect. Such variability may arise from dependent effect sizes, as the same participants often contribute to multiple measures within a study, and from potential moderators, such as text type or post-editor characteristics. To address these sources of heterogeneity, the current study employs a three-level meta-analytic approach. This model accounts for within-study dependence, measurement error, and between-study variability.

Conventional meta-analysis approaches, such as averaging effect sizes and selecting one effect size per study, are frequently utilized to circumvent the problem of dependence among effect sizes (Mathias et al., 2021). Nevertheless, these ad hoc approaches potentially lead to the forfeiture of opportunities to fully exploit all accessible data for the purpose of addressing pertinent research questions (Cheung, 2019). Compared with traditional methods, three-level meta-analysis addresses sample and measurement errors within single studies. It also allows the examination of heterogeneous factors across studies by integrating multiple effect sizes (Mathias et al., 2021). As a result, to fill the aforementioned gaps and provide a comprehensive understanding of the strength of the relationship between MTPE and the post-editor’s cognition, as reported previously, a three-level analysis will be conducted in the present study.

### 1.1. Theoretical Foundations of the Link Between Post-Editing and Cognition

The priming effect theory and the construction–integration model (CIM) are used to explain cognitive activities observed in machine translation post-editing (MTPE). Rather than providing task-specific predictions, these theories focus on how prior linguistic input shapes subsequent cognitive processing. First, the priming effect is a well-documented phenomenon in which prior stimuli influence the perception and processing of subsequent input (Solso et al., 2005). This effect can alter cognitive processing strategies during task performance (Young & Jennings, 2022) and is commonly distinguished into positive and negative priming (Tipper, 1985). In MTPE, positive priming may increase processing fluency and speed. However, it can reduce vigilance and increase the likelihood of overlooked translation errors, particularly in neural machine translation post-editing (NMTPE, Castilho et al., 2017; X. Wang et al., 2024). By contrast, negative priming can slow processing and increase cognitive effort, making error correction more difficult and sometimes leading to inappropriate revisions (Yamada, 2019; Nitzke et al., 2019). These priming mechanisms illustrate how prior machine-generated output can shape post-editors’ cognitive engagement across different MT paradigms.

According to the construction–integration model (CIM; Kintsch, 1988), text comprehension and production involve creating a coherent mental representation, where propositions are connected and integrated with prior knowledge (Kintsch & van Dijk, 1978). During post-editing, error detection and correction rely on the interaction between newly processed information and existing cognitive representations. They also depend on the strength of semantic and pragmatic links within the evolving text base. Although empirical studies directly applying the CIM to MTPE are limited, research on text comprehension and translation cognition supports its relevance.

Moreover, exposure to pre-translated text can influence attention allocation, self-monitoring, and revision behavior. While it often reduces processing effort, it may simultaneously increase the risk of overlooked errors (O’Brien, 2011; Elming et al., 2014). Under certain conditions, however, MT input can enhance self-monitoring and alleviate comprehension-related difficulties (Mangalath, 2010; Oh, 2022). These studies suggest that the CIM provides a mechanistic account of cognitive resources in comprehension and monitoring during MTPE, rather than serving to predict the effects of specific moderators. This perspective aligns with resource-based views of cognition, which conceptualize post-editing as the allocation of limited cognitive resources under varying task and text conditions (Kahneman, 1973). In this context, the priming effect theory and the CIM are suitable theoretical tools for explaining the relationship between MTPE and post-editors’ cognition.

### 1.2. Moderating Variables of Post-Editing and Cognition

Drawing on Meng et al. (2024), this study conducted a literature review to identify moderators. First, Reiss (1981) proposed a text typology comprising informative, expressive, and operative texts. These types convey different text norms (Reiss & Rhodes, 2000) and thus impose varying cognitive challenges. Scholars generally agree that expressive texts usually demand more cognitive effort than informative or operative texts, as they require translators to allocate greater mental resources to uphold comparatively high-quality translations (e.g., Jia et al., 2019; Y. Wang & Daghigh, 2024).

In addition, language divergences across texts merit attention. Chinese and English belong to distinct language families (Lyle & William, 2008). Differences in grammar, part-of-speech change, and morphology may yield heterogeneous MTPE findings, even under identical participants or designs. For example, Huang and Carl (2022) observed increased cognitive effort to identify translation difficulties in English–Chinese MTPE, whereas Daems et al. (2017a), using English–Dutch texts, reported opposite patterns. These disparities highlight the need to examine underlying factors, yet empirical evidence on how language families shape cognition remains limited. Moreover, despite hypotheses that translation direction reallocates cognitive resources (Altarriba & Basnight-Brown, 2007), no empirical work has verified its causal effects in MTPE. Hence, this study considers language families and directions as moderators. MT systems also warrant consideration, as different systems produce distinct error types (Daems et al., 2015) affecting cognition. Prior research links coherence, meaning shifts, and structural errors to cognitive demands across SMT, NMT, and AMT (e.g., Daems et al., 2017a; Vardaro et al., 2019). System-level differences in architectures, engines, and evaluation metrics likewise influence the amount of cognitive effort required. This amount of cognitive effort reflects the cognitive resources translators must allocate to manage errors and maintain output quality (e.g., Rivera-Trigueros, 2022). For instance, Gutiérrez-Artacho et al. (2018, 2019) found that ProMT (SMT-based) yielded higher-quality output and lower cognitive effort than Systran (NMT-based) for translators. Therefore, MT systems are included as moderators in this study.

Drawing on Bloom’s (1956) cognitive domains and prior research, post-editor cognition is categorized into five dimensions: cognitive effort, load, demand, memory, and attention. Among these, cognitive effort reflects the number of mental resources allocated to ensure translation quality. This metric always functions both as a diagnostic indicator of task engagement and as a necessary contributor to successful post-editing (Huang & Carl, 2022; Kahneman, 1973). Cognitive load and demand capture the external task and text-related cognitive requirements, reflecting the processing cost imposed by translation challenges (Sweller et al., 2011). Memory refers to the working memory processes necessary to maintain and integrate information during post-editing (Baddeley & Hitch, 1974). Attention reflects the allocation of focus and monitoring of text segments, supporting error detection and comprehension (Brothers et al., 2022). These five dimensions provide a coherent framework to interpret the observed effects of MTPE on post-editors’ cognition.

Eye-tracking devices also influence cognitive measurement. Their resolution and sampling rate differences can lead to divergent findings. Huang and Carl (2022) used Tobii X2–60 (60 Hz), whereas Cui et al. (2023) employed Gazepoint GP3 HD (60–150 Hz), and despite comparable metrics, their results differed markedly.

Turning to individual factors, whether a study reported the post-editor’s attitude towards PE was associated with differences in observed effect sizes. For example, Qian et al. (2022) found that studies reporting PE attitudes showed lower effect sizes, a pattern also observed in Guerberof Arenas (2008) and Vieira (2015). Professional experience also plays a role. De Almeida and O’Brien (2010) found that higher expertise predicts faster performance and reduced cognitive effort. However, Balling and Carl (2014) and Lu and Sun (2018) observed minimal effects of experience. Gender has also been associated with cognitive differences (Kheloui et al., 2023). Based on these findings, the present study includes post-editor attitude, professional experience, and gender as moderator variables.

In summary, the present study explores these moderator factors: text types, language families, translation directions, post-editors’ attitudes towards PE, MT error types, cognitive types, MT systems, eye-tracking devices for sampling, post-editors’ experience, and gender. All of these factors are considered as categorical variables.

### 1.3. Current Study

This study follows the PRISMA 2020 (Page et al., 2021) guidelines and offers the first three-level meta-analysis examining the relationship between post-editors’ cognition and MTPE, thereby contributing new empirical evidence to translation cognition research. Guided by the overall research framework, we first summarized effect sizes and categorized variables reported in previous studies. We then distinguished variables related to editors’ cognition, MTPE, and their potential moderators. Subsequently, each variable was identified and assigned to relevant subcategories, following the established heuristic that variable differentiation is essential in meta-analytic reviews (Schneider & Preckel, 2017). Drawing on theoretical foundations and research hypotheses, we classified ten categories of potential moderators to capture the full range of interactions between MTPE and editors’ cognition.

Methodological quality was also carefully considered. Following Polanin et al. (2019), we adopted the systematic criteria proposed by Shea et al. (2009), which facilitate the structured categorization and analysis of moderators in meta-analyses. In accordance with these standards, we provide a clear account of analytic procedures, the validity and robustness of results, and other essential methodological details. By identifying characteristics that influence effect sizes, the present study enhances overall methodological rigor. We expect that future researchers can adopt comparable procedures to conduct similarly high-quality meta-analyses, building on the methodological insights offered here.

To sum up, this study is designed to address the following four questions:

RQ1: Does a relation exist between MTPE and the post-editor’s cognition?

RQ2: If a relationship between them is identified, what is the strength and direction of this relationship?

RQ3: What are the moderating factors between MTPE and the post-editor’s cognition?

RQ4: What is the significance, the strength of the relationships, and the directions of these moderating factors?

## 2. Methods

### 2.1. Literature Search and Eligibility Criteria

To capture the breadth of research in this area, our search covered journal articles, conference papers, and dissertations. We systematically queried five databases, including Web of Science (WOS), ScienceDirect (SD), China National Knowledge Infrastructure (CNKI), ProQuest (PQ), and Scopus, using targeted terms consistent with the quantitative nature of meta-analysis. For example, in PQ, we utilized the following search query: (“post-editing”) AND (“eye-tracking” OR “eye movement” OR “gaze movement” OR “gaze tracking”) AND (“machine translation”). Searches were restricted to titles, abstracts, authors, publication years, and sources and were completed on 28 November 2024.

Following Polanin et al.’s (2019) guidelines, two trained raters conducted the screening. We subsequently performed a manual cross-reference check to minimize missed studies. In total, 613 records were retrieved. In accordance with PRISMA 2020 (Page et al., 2021), studies were included only if they met the following criteria:

- Reported at least one effect size linking an MTPE variable to a cognitive variable, as conceptualized above;
- Provided effect sizes derived from quantitative data rather than case studies;
- Reported sufficient statistics to calculate effect sizes when not directly provided;
- Examined cognitive outcomes of post-editors;
- Were peer-reviewed with unambiguous data;
- Provided full text in a specific language and clearly stated the experimental language.

**Figure 1:**
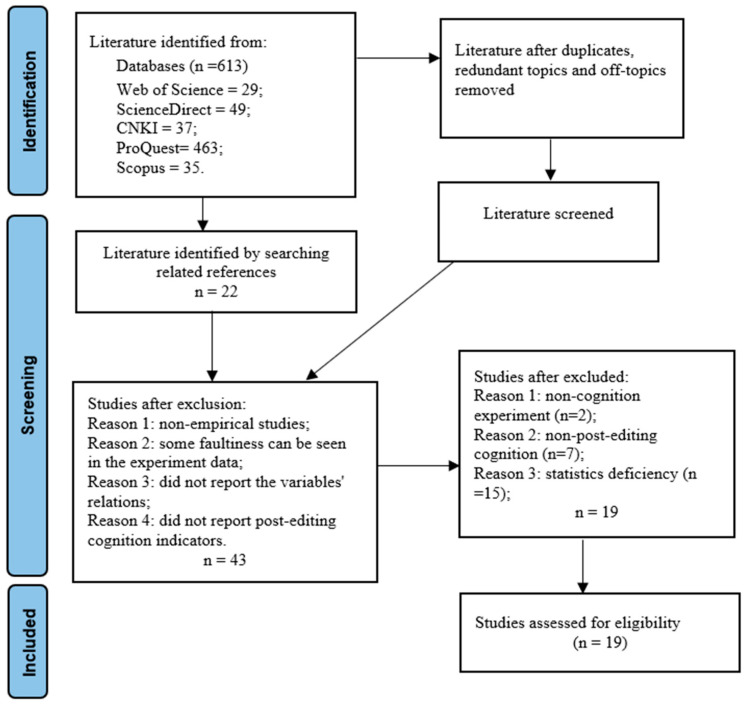
PRISMA flowchart summarizing database search and report screening.

After removing duplicates and topic redundancies, we applied these criteria to screen the literature. Ultimately, 19 studies were retained, yielding 193 effect sizes. Figure 1 summarizes the search and selection procedure.

### 2.2. Variable Coding

The properties of all meta-analytic studies meeting the inclusion criteria were coded according to explicit criteria. Each study was independently coded by two authors based on the following attributes: (a) first author; (b) publication year; (c) sample size; (d) gender distribution; (e) average age; (f) participant type (professional or student translators); (g) text type (informative, expressive, operative); (h) translation direction (L1→L2 or L2→L1); (i) language family (primarily Indo-European); (j) reported attitudes toward PE; (k) MT system used (e.g., Google, Systran, DeepL); (l) reported MT error types; (m) cognitive type (load, demand, memory, effort, attention); and (n) cognitive measurement tools (e.g., Eyelink 1000, Tobii T60).

Three principles guided the coding. First, each independent sample was coded once; when studies reported multiple independent samples, each was coded separately. If a study did not provide a sample size for an experimental group, we adopted Quarmley et al.’s (2022) method and calculated group size by dividing the total sample by the number of groups. Second, each variable indicator was coded when multiple indicators were reported. Third, for longitudinal studies, only the initial measurement was coded. Inter-rater reliability, computed using Cohen’s kappa, reached 0.881, indicating substantial agreement. Any discrepancies were resolved through joint review.

Study quality was assessed using the NIH Quality Assessment Tool for Observational Cohort and Cross-Sectional Studies (National Institutes of Health, 2014). Each criterion met was scored as “1”, whereas criteria rated as “No” or “NA” received a “0”. Total scores classified studies as good (above 7), fair (5–7), or poor (below 5). Only studies rated at least “fair” were retained for the meta-analysis to ensure methodological robustness.

### 2.3. Calculation of Effect Sizes

In all included studies, the correlation coefficient (r) between MTPE and the post-editor’s cognition, or their subcategories, was used as the effect size. When r was not reported directly, it was derived from available statistics, including mean (M), standard deviation (SD), sample size (n), degrees of freedom (df), t, F, or χ^2^ values. Effect sizes were classified as small, medium, and large following Cohen’s (1992) standards, corresponding to r = 0.20, 0.50, and 0.80, respectively. The formulas used for conversions were as follows (Zheng & Peng, 2001; Borenstein et al., 2021):

(1) $$t=\frac{\bar{x}-\mu }{SD/\sqrt{n}}$$

(2) $$r=\sqrt{\frac{t^{2}}{t^{2}+df}}$$

(3) $$r=\sqrt{\frac{F}{F+df}}$$

(4) $$r=\sqrt{\frac{X^{2}}{n_{1}+n_{2}}}$$

Notably, in Equation (1), $\bar{x}$ represents the sample mean. All included studies were independent single-sample designs, and μ is the assumed total mean calculated as the average of M across studies. In Equations (2) and (3), df = n − 1, corresponding to the independent one-sample design. In our dataset, 193 effect sizes were included, of which 20 were originally negative. Following Zheng and Peng (2001), squared statistics (t^2^, F, χ^2^) were converted to r. This conversion reflects the effect size magnitude but does not retain the original direction. Because this conversion discards directionality, we conducted a sensitivity analysis by removing the 20 negative effect sizes and recalculating the pooled effect. The pooled r changed only slightly, from 0.474 (95% CI [0.301, 0.648]) to 0.434 (95% CI [0.272, 0.573]), indicating that discarding directionality in the original conversion did not materially affect the overall conclusions. Within-subject and between-subject designs were not separately adjusted because this meta-analysis operates at the study level using three-level modeling. Each study contributed at least one effect size, and the model accounts for dependencies among multiple effect sizes within the same study. Consequently, all effect sizes represent study-level correlations while appropriately handling within-study and between-study variance. This procedure ensured that all effect sizes were calculated consistently and transparently across studies. The dataset generated in the study was stored in an international database, Mendeley Data (doi: 10.17632/vtnnm68zr5.1).

### 2.4. Three-Level Meta-Analysis Model

In conventional meta-analysis, effect sizes were assumed to be independent of one another. Thus, an effect size was calculated from each study (Assink & Wibbelink, 2016). However, a considerable number of source studies reported more than one effect size from the same sample, suggesting correlations among them. Traditional meta-analysis neglects these correlations, leading to an overestimation of overall effect sizes (Lipsey & Wilson, 2001). By contrast, three-level meta-analysis can handle these dependent effect sizes, maximizing the retention of information from the original study to enhance statistical validity. Consequently, based on a random-effects model in three-level meta-analysis, publication bias, main-effect tests, heterogeneity tests, and moderator analyses are conducted in the subsequent sections. Note that these analyses are performed using the metafor and metaviz packages (Viechtbauer, 2010) in R x64 4.1.1 software, following the methods described by Assink and Wibbelink (2016).

### 2.5. Publication Bias, Heterogeneity and Moderator Analyses

Publication bias arises when studies with small or non-significant effects are less likely to be published (Thornton & Lee, 2000). To assess this file drawer effect, we inspected the asymmetry of the power-enhanced sunset funnel plot and conducted Egger’s regression test (Egger et al., 1997). The sunset funnel plot, which incorporates color-coded power regions and a secondary power axis, enables a power-sensitive evaluation of small-study effects (Kossmeier et al., 2020). A non-significant Egger’s test (*p* > 0.05) suggests negligible publication bias, whereas a significant result (*p* < 0.05) or visibly low-powered effects in the funnel plot prompt the use of the trim-and-fill procedure. If trimming and filling do not materially alter the results, publication bias is considered minimal (Duval & Tweedie, 2000).

In the three-level meta-analysis, we estimated variance at three sources: sampling variance (Level 1), within-study variance (Level 2), and between-study variance (Level 3) (Cheung, 2014). Overall heterogeneity (Level 1) was tested using the Q-test, and Level 2 and Level 3 heterogeneity were examined using one-tailed log-likelihood ratio tests. Following Higgins et al. (2003), I^2^ values of 25%, 50%, and 75% were used as benchmarks for small, medium, and large heterogeneity. Moderator analyses were then conducted to identify sources of variability. Consistent with Hox et al. (2017), we did not pool significant moderators in a multiple meta-regression because the individual study samples were too small to support reliable multivariate modeling.

## 3. Results

### 3.1. Included Studies Characteristics and Quality Assessment

We included 19 studies, 193 effect sizes, and 492 subjects, with a time span from 2014 to 2024. The number of effect sizes in each study ranged from 1 to 22. Among the included studies, five were openly published Chinese articles, and 14 were English articles. No grey literature, such as grey articles, reports, conference papers, or dissertations, was included. The specific details can be found in Table 1. Regarding the quality assessment of the included studies, their scores ranged from 6 to 9 (see Table 1), with an average score of 8.158, which is higher than the theoretical mean (7 points). Thus, these studies illustrate satisfactory quality and validity to run the three-level meta-analysis.

### 3.2. Publication Bias Analysis

**Figure 2:**
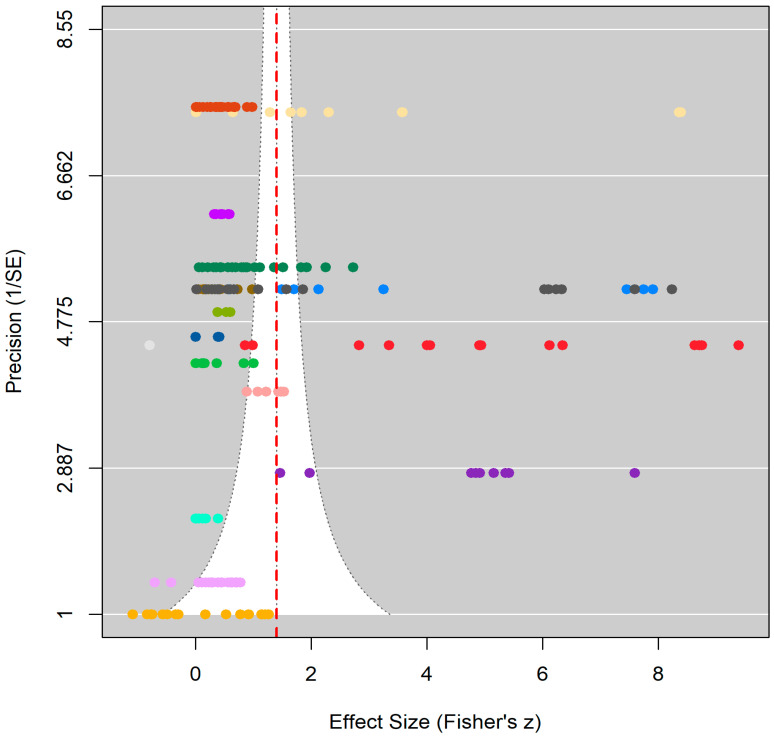
Funnel plot of publication bias.

A visual inspection of the funnel plot shows that the included studies are distributed relatively symmetrically around the overall effect size (overall effect = 1.398, 95% CI [0.582, 2.214]; see Figure 2). This visual symmetry suggests a low likelihood of substantial publication bias. Most studies cluster near the center of the plot. This pattern reflects moderate to high precision, ranging from 1.414 to 7.55. Precision is inversely related to standard errors. A few studies have lower precision, as indicated by higher standard errors up to 0.707. These lower-precision studies do not appear to skew the overall distribution. In a nutshell, the distribution of effect sizes and their precisions provides supportive evidence for the meta-analytic estimate. It indicates that the observed overall effect is reasonably reliable.

Egger’s regression test unveils a non-significant effect of bias (t = 0.7076, *p* = 0.4800 > 0.05, b = 1.2951, df = 191, 95% CI [0.5029, 2.0873]). The two findings indeed confirm the absence of publication bias among the included studies. Consequently, the trim-and-fill method is unnecessary for adjusting publication bias, as it exerts minimal influence on our meta-analysis. Additionally, the leave-one-out sensitivity analysis indicated that the overall effect was not substantially influenced by any individual effect size. The estimated overall effect ranged from 0.4648 to 0.4846, consistently within the 95% CI of the full model. This demonstrates the robustness of the main findings.

### 3.3. Main Effect and Heterogeneity Analysis

A positive correlation was observed between the overall effect size of MTPE and the post-editor’s cognition (r = 0.474, df = 192, se = 0.0880, t = 5.394, *p* < 0.0000001, 95% CI [0.301, 0.648]). This finding offers a more comprehensive response to RQ1 and RQ2. According to Cohen’s (1992) assertion, a correlation coefficient of 0.474, which exceeds 0.40, indicates a large effect size. This association indicates that MTPE is linked to higher levels of cognitive processing during post-editing activities, rather than implying an enhancement in cognitive efficiency or performance.

To assess heterogeneity at Level 1, we employed the Q-test for overall variance. The test yielded a statistic of 1904.036 (*p* < 0.00000001, I^2^ = 6.829%). To account for the heterogeneity at Level 2 and Level 3, one-sided likelihood ratio tests were performed. At Level 2, medium heterogeneity (σ^2^ = 0.182, *p* < 0.05, I^2^ = 20.298%) was observed within the study, contributing 20.298% to the overall variance. In contrast, at Level 3 (σ^2^ = 0.361, *p* < 0.0001, I^2^ = 79.702%), substantial heterogeneity between studies was demonstrated, in line with the findings of Higgins et al. (2003). Given this, a moderator analysis is essential to determine how MTPE impacts the post-editor’s cognition.

### 3.4. Moderator Variables

It is important to note that the variable “reported PE attitudes” reflects a methodological characteristic at the study level rather than translators’ psychological attitudes. The overall moderator test for this methodological variable (F = 4.124, *p* = 0.044) indicates that the reported vs. non-reported distinction accounts for some heterogeneity across studies (see Table 2). Specifically, effect size estimates differed between studies that reported PE attitudes and those that did not. Studies without reported PE attitudes showed a positive correlation (r = 0.534, k = 181, 95% CI [0.363, 0.705]), whereas estimates from studies reporting PE attitudes were not significantly different from zero (r = −0.031, k = 12, 95% CI [−0.553, 0.491]). These findings likely reflect methodological differences at the study level rather than true cognitive effects of translators’ attitudes.

A similar pattern was observed for text types. While the omnibus F-test for text type was not significant (F = 0.822), indicating that text type as a moderator does not explain heterogeneity across studies, individual levels showed effect sizes significantly different from zero. For informative texts, r = 0.400 (k = 128, *p* < 0.001, 95% CI [0.190, 0.611]); for expressive texts, r = 0.642 (k = 53, *p* = 0.018, 95% CI [0.113, 1.172]); and for operative texts, r = 0.668 (k = 80, *p* = 0.003, 95% CI [0.233, 1.103]).

Regarding measurement tools and cognitive types, Eyelink (r = 0.459, k = 55, *p* = 0.014, 95% CI [0.092, 0.825]) and Tobii (r = 0.509, k = 61, *p* = 0.002, 95% CI [0.196, 0.823]) showed significant positive correlations with post-editor cognition, while Gazepoint (r = 0.574, k = 25, *p* = 0.051) and others (r = 0.372, k = 52, *p* = 0.056) were marginally significant. The omnibus F-test for measurement tools (F(3,189) = 0.151) indicates that the differences among tools do not significantly explain heterogeneity. Similarly, cognitive effort (r = 0.551, k = 128, *p* < 0.001, 95% CI [0.314, 0.788]) was significant, but the omnibus F-test across cognitive types (F (4,188) = 0.395) was not. Overall, aside from the factors mentioned above, no other significant moderators were identified in the present study.

## 4. Discussion

### 4.1. Positive Link Between Machine Translation Post-Editing and Post-Editors’ Cognition

Our first key finding is that MTPE is positively correlated with post-editors’ cognition, with a large effect size observed in a context free from publication bias. The magnitude of the effect size reflects the consistency of associations across heterogeneous cognitive indicators. At the process level, this association may suggest that translators expend more time and bear higher cognitive costs in order to complete the rendition. This finding is consistent with previous studies (Moorkens et al., 2018; Vardaro et al., 2019; Huang & Carl, 2022; X. Wang et al., 2024). This heightened cognitive cost may be associated with higher error rates in contemporary MT systems. This is particularly the case for systems based on SMT algorithms. These systems may still produce substantial errors despite being trained on large-scale parallel corpora (Benko et al., 2024). Over time, sustained reliance on MTPE may be associated with changes in human translation competence (Pshenichnikov, 2024). It is possible that reduced translation competence could prompt translators to allocate additional cognitive resources to correcting low-quality pre-translated texts, potentially affecting translation productivity.

Hence, numerous scholars have posited that training top-notch MT systems requires large-scale, high-quality corpora. However, human-labeled corpora often contain latent errors and may overlook figurative language, which can cause MT systems to misinterpret nuanced expressions. As a result, post-editors must engage in extrinsic load management, first decoding the original figurative intent and then correcting machine-generated errors. According to the negative dimension of the priming effect theory, cognitive expenditure during the priming phase may be higher when participants engage in this dual-task process (S. Wang et al., 2024).

Apart from the aforementioned aspects, eye-movement measures have been widely used to infer cognitive processing during MTPE tasks. According to the eye–mind assumption (Just & Carpenter, 1976), fixation-based measures reflect moment-to-moment processing demands, whereas other eye-tracking metrics index complementary cognitive subprocesses. Thus, eye-tracking metrics cannot be treated as interchangeable proxies for “cognition”. For example, during text modification, fixations and pauses indicate increased processing difficulty or local comprehension disruptions, while saccades and regressions reflect attentional shifts and integrative processing (Rayner, 1998). In the context of MTPE, these distinct indicators are particularly apparent during the initial phase of post-editing, when post-editors adapt to and compare the MT output. In this stage, pauses and hesitations frequently occur. These phenomena are often accompanied by increases in pupil diameter, which easily leads to heightened cognitive load under increased processing demands (Holmqvist et al., 2011). Such changes suggest that PE texts may disrupt the translator’s cognitive rhythm, as manifested in altered pause patterns and fixation distributions (O’Brien, 2006).

Subsequently, the post-editor enters a decision-making stage that requires the renewed allocation of cognitive resources, including effort, load, and attentional control. At this stage, the editor evaluates alternative target-language expressions and determines whether to retain, modify, or reject machine-generated output. For terminologies characterized by higher morphological or domain-specific complexity, this decision-making process becomes particularly demanding. According to the CIM, such decisions may reflect the integration of prior knowledge with newly processed information. This process mobilizes multiple cognitive operations rather than a single mechanism.

In a nutshell, the foregoing discussion illustrates that post-editing cognition is shaped by multiple interacting factors. In line with this complexity, significant variance is observed at both Level 2 and Level 3, indicating that the heterogeneity in the main effect cannot be examined in isolation (Mathias et al., 2021). Beyond the general cognitive mechanisms outlined above, it is therefore necessary to examine potential moderators to account for this variability.

### 4.2. Moderators of Machine Translation Post-Editing to Post-Editors’ Cognition

In the case of reported vs. non-reported PE attitudes, the moderator reflects a methodological distinction between studies rather than the valence of translators’ attitudes. For the three text types, each showed a significant effect within its level, but the omnibus test was not significant, indicating that text type does not account for heterogeneity across studies. These level-specific effects provide partial support for previous assertions (Krings, 2001; Sánchez-Gijón et al., 2019; Yamada, 2019; Y. Wang & Daghigh, 2024). For measurement tools, certain devices, such as Eyelink and Tobii, showed significant level-specific effects, indicating that cognition measured with these tools correlates with MTPE. These findings may have several possible explanations.

First, whether a study explicitly measured translators’ attitudes toward post-editing reflects methodological choices at the study level rather than the cognitive effects of translators’ attitudes. This is because studies that measured attitudes often differed in design features, participant populations, or MT systems, and these differences could not be disentangled within the current coding scheme. Prior research (De Almeida, 2013; Daems et al., 2017a, 2017b; Qian et al., 2022) has shown that differences in study design and measurement protocols, including the use of attitude measures, shape post-editors’ cognitive outcomes. As Sweller et al. (2011) noted, introducing extra measures may increase extraneous load by adding task demands and reflective processing requirements, thereby influencing participants’ cognitive engagement. Meanwhile, prior exposure to reflective tasks or additional questionnaires may prime post-editors to allocate attention differently.

Second, although text types do not explain heterogeneity across studies, they differ in terminology complexity, cultural context, and stylistic features, which can pose varying translation challenges. Addressing these challenges requires post-editors to integrate new information with prior knowledge and engage reasoning processes, increasing cognitive effort. This effort may manifest in prolonged fixations, saccades, or changes in pupil dilation, reflecting the cognitive demands of the task rather than any moderating effect of text type. For instance, translating a descriptive prose piece may require more interpretive and creative processing than fact-based reporting, yet in both cases, cognitive load rises due to complex information integration and task demands.

Third, regarding measurement tools, this study provides the first statistical evidence for a positive relationship between eye-tracking instrumentation and post-editors’ cognition. Devices such as Eyelink, Tobii, Gazepoint, and others are commonly used to monitor cognitive shifts during post-editing. Differences in sampling rate, visual angle accuracy, and invasiveness directly affect the precision of cognitive monitoring, as more accurate measurements are better able to capture variations in attentional allocation and cognitive changes. These findings echo the divergence in cognitive outcomes reported by Huang and Carl (2022) and Cui et al. (2023). Specifically, higher sampling rates (e.g., 120 Hz) allow for the detection of fine-grained eye movements, such as microsaccades and saccadic suppression, which reflect nuanced cognitive rhythms. In contrast, less precise or more invasive equipment tends to introduce measurement noise, which may artificially attenuate observed levels of cognitive engagement. This explanation is compatible with the construction–integration model (CIM), as more precise measurement enables finer-grained observation of the conceptual integration processes involved in post-editing.

Lastly, the number of effect sizes differed across cognitive types. Only cognitive effort showed a significant positive effect within its level. Other cognitive types, including cognitive memory (k = 6) and cognitive demand (k = 18), were based on comparatively small subsamples. These estimates should therefore be regarded as exploratory and interpreted with caution. The significant effect observed for cognitive effort suggests that increased cognitive effort may represent a necessary and diagnostically informative allocation of cognitive resources during post-editing. Moreover, the non-significant omnibus test indicates that cognitive type does not explain heterogeneity across studies. Although these cognitive types did not account for between-study heterogeneity, the within-level effect for cognitive effort warrants cautious theoretical consideration. Specifically, the pattern observed for cognitive effort aligns with theoretical expectations from Bloom’s (1956) theory framework and the information-processing model proposed by Atkinson and Shiffrin (1968). Drawing on Bloom’s knowledge integration framework, cognitive effort may play a pivotal role in knowledge creation and synthesis. It may function as a guiding mechanism that coordinates the deployment of other cognitive resources. In a parallel sense, the information-processing model suggests that cognitive effort may serve as an index of overall processing expenditure. These theories converge in highlighting the role of cognitive effort as a potential mediator in post-editing actions (Krathwohl, 2002).

Apart from reported vs. non-reported PE attitudes, the remaining variables did not show significant omnibus effects, indicating that they do not function as moderators. Nevertheless, some level-specific effects or trends were observed, which are discussed below. At the participant level, gender differences may be constrained by cultural–pragmatic factors and preferred translation strategies. Translation experience also shows no significant effects. This may be due to the use of external resources, such as dictionaries, or to the non-routine nature of the tasks. At the task and language level, translation direction and language family diverge from earlier findings (Jääskeläinen, 1999; Baker, 2018; Abu-Rayyash & Alhawamdeh, 2024). This divergence may reflect the complex and non-linear interactions among linguistic and cognitive processes within translation systems. At the technological level, MT system effects were often not systematically controlled or reported, which may account for their non-significant results. Finally, residual cognitive types and error types showed no significant associations. These null effects may be influenced by contextual factors, including laboratory settings, task duration, and the relative uniformity of errors in news texts. This phenomenon highlights contextual limits on cognitive effects.

### 4.3. Contributions and Limitations

This study offers several novel contributions to the understanding of MTPE and post-editor cognition. From a theoretical perspective, this meta-analysis aligns with the interdisciplinary translation process research framework proposed by Zhang (2020), which emphasizes examining translation cognition in technologically mediated environments such as machine translation post-editing. Our study also serves as a practical manifestation in the era of machine translation. So, by synthesizing prior findings, it shows that both negative and positive priming effects are consistently associated with cognitive patterns observed during post-editing. They also suggest that pre-translated texts are associated with changes in post-editors’ cognitive engagement that are compatible with the priming effect theory. In this vein, negative priming provides a plausible interpretation for increased cognitive demands associated with error detection and revision, whereas positive priming offers a theoretical lens for understanding how exposure to pre-translated input may, under certain task conditions, be associated with reduced cognitive effort. These interpretations extend current discussions on how exposure to pre-translated input may interact with cognitive processing during post-editing while highlighting the need for future meta-analyses that explicitly code the content and valence of translators’ attitudes.

Furthermore, this study contributes by examining associations between MTPE and multiple cognitive types across heterogeneous indicators. By distinguishing cognitive effort, load, memory, demand, and attention, the meta-analysis highlights that different cognitive dimensions relate to post-editing in distinct ways. These patterns provide integrative evidence that is consistent with the CIM, insofar as post-editing involves the allocation and coordination of cognitive resources during comprehension, monitoring, and revision. These results suggest that the CIM provides a useful lens for understanding cognition in modern MT paradigms, such as NMT and multimodal MT. This is because these paradigms rely on deep learning architectures, including recurrent neural networks, multi-layer attention mechanisms, and repeated conceptual integration. However, it should be noted that the link between CIM and the observed cognitive patterns is inferential, based on the meta-analytic evidence rather than direct empirical testing. Overall, the results suggest that finer-grained cognitive distinctions are necessary for refining theories of translation cognition.

From a practical perspective, this study advances MTPE practice by providing evidence-based guidance for training and experimental design. Monolingual corpora can be integrated to improve MT robustness, volume, and quality. This is particularly important for minority languages, where generating pseudo-bilingual data can enhance language structures. Post-editor training should prioritize text types according to their cognitive relevance. Informative texts should come first, followed by expressive texts, and then operative texts. This approach helps post-editors develop context-sensitive editing skills and compensate for semantic or emotional scarcity. The identified moderators offer actionable guidance for designing training tasks and auxiliary measures, such as questionnaires and interviews, when assessing post-editors’ cognition. For eye-tracking research, instruments should balance temporal resolution with specific experimental goals. Higher sampling rates (e.g., 250–500 Hz or above) allow the capture of fine-grained cognitive patterns, but the optimal choice depends on the metrics of experimental design and practical constraints. Applying these evidence-based practices can enhance the reliability and effectiveness of MTPE training and evaluation.

Despite these contributions, several limitations should be acknowledged. First, a three-level meta-analytic model was adopted to account for dependency among effect sizes. However, some moderator categories included relatively small numbers of effect sizes. Accordingly, findings related to these moderators should be interpreted cautiously and regarded as exploratory rather than confirmatory. Future studies with larger and more balanced datasets are needed to substantiate these patterns. Second, the operationalization of cognitive constructs was constrained by the measures reported in the primary studies. Although cognitive load, effort, and memory were distinguished, variability in instruments and experimental designs may have introduced measurement noise, highlighting the need for more standardized and ecologically valid tools in MTPE cognition research. Third, this meta-analysis relied on aggregated study-level data rather than individual-level data. Consequently, fine-grained cognitive dynamics at the process level could not be directly examined. Future research combining meta-analytic evidence with process-tracing methods may provide a more comprehensive understanding of post-editors’ cognition. Finally, most included studies were conducted in controlled experimental settings. While this enhances internal validity, it may limit the generalizability of the findings to professional and real-world post-editing contexts. Further research in authentic workplace environments is therefore warranted.

## 5. Conclusions

Understanding how MTPE is associated with post-editors’ cognitive processes is of paramount importance to the translation service industry in the era of large language models. Such understanding is fundamental to a range of crucial aspects, including informing the training and quality development of MT systems, research on translation-related human intelligence, and the interpretation of cognitive patterns during translation tasks. The results of the present study make a positive contribution to clarifying the relationship between MTPE and post-editors’ cognitive measures. They highlight systematic variability associated with study-level characteristics, including whether PE attitudes were reported, eye-tracking methods, text characteristics, and types of cognitive indicators. Despite these limitations, the findings provide an empirical foundation for future research on translation cognition in MTPE contexts. Future efforts should address the aforementioned limitations. This includes improving experimental design control, increasing sample sizes, and testing multivariate models. These steps are expected to support more refined moderator analyses in subsequent MTPE and cognition studies.

## Figures and Tables

**Table 1:** Basic information on the characteristics of the included studies.

Author (Year)	SS	TE	TT	TD	LP	PA	MT Systems	ET	CT	Measurement Tools	QAS
Qian et al. (2022)	41	S&P	I	L2–L1	EC	R	Youdao and Sogou	R	attention	EyeLink Portable Duo	8
X. Wang et al. (2021)	30	S	O	L2–L1	EC	NR	Google	NR	load	EyeLink 1000 Plus	9
Lu and Sun (2018)	30	S	I&E&O	L1–L2	CE	NR	Other	NR	effort	Tobii TX300	9
X. Wang et al. (2024)	11	S	E	L2–L1	EC	NR	DeepL	NR	effort	Other	8
Zhong et al. (2024)	30	S	O	L1–L2	CE	NR	Google and ChatGPT	R	load	Tobii S1200	8
Cui et al. (2023)	33	S	I&E&O	L2–L1	EC	NR	Google	NR	effort	Gazepoint GP3 HD Desktop Eye Tracker	9
Alves et al. (2016)	21	S	I	L2–L1	EP	NR	Other	NR	effort	Tobii T60 eye tracker	7
Fonseca (2019)	59	S&P	I	L2–L1	EP	NR	Google	NR	effort	Tobii T60 eye tracker	7
Daems et al. (2017a)	23	S&P	I	L2–L1	ED	R	Google	NR	load	EyeLink 1000	8
Huang and Carl (2022)	21	S	E	L2–L1	EC	NR	Other	NR	effort	Tobii X2–60 eye tracker	6
Jia and Zheng (2022)	60	S	I	L2–L1	EC	NR	Google and Systran	NR	effort	Eyelink 1000 plus	9
Lacruz et al. (2014)	5	S	O	L2–L1	SE	NR	Other	NR	demand	Other	8
Yang et al. (2023)	24	S	I	L1–L2	CE	NR	Google	NR	load	Other	9
Y. Wang and Daghigh (2024)	24	S&P	I&E&O	L1–L2	CE	NR	Google	NR	effort	Tobii Pro Fusion eye tracker	8
Lacruz and Shreve (2014)	4	S&P	I	L2–L1	SE	NR	Google	NR	effort	Other	8
Lourenço da Silva et al. (2017)	18	P	I	L2-L1&L1-L2	CP	NR	Google	NR	effort	Tobii T120 remote eye tracker	8
Vardaro et al. (2019)	27	P	I	L2–L1	EG	NR	Other	NR	effort	SMI RED250 Mobile eye tracker	9
Daems et al. (2017b)	23	S&P	I	L2–L1	ED	NR	Google	R	effort	EyeLink 1000 eye tracker	9
Sánchez-Gijón et al. (2019)	8	P	O	L1–L2	EP	NR	Other	NR	memory	Other	8

Note: As for abbreviation, Year = publication year; TE = translation experience; SS = sample size; S = student, P = professional translators; TT = text type; I = informative texts, E = expressive texts, O = operative texts; TD = translation direction; LP = language pair; CE = Chinese–English, EC = English–Chinese, EP = English–Portuguese, ED = English–Dutch, SE = Spanish–English, CP = Chinese–Portuguese, EG = English–German, PA = Post Attitude, R = reported, NR = non-reported; ET = error type; CT = cognitive type; MTs = measurement tools. QAS = quality assessment score.

**Table 2:** Moderator analysis.

Moderator	k	r	95% CI	Omnibus F	Level *p*
Gender	0.525				
Female	98	0.409	[0.161, 0.657]	-	0.470
Male	98	0.409	[0.161, 0.657]	-	0.470
Translation Experience	0.110				
Student	179	0.498	[0.262, 0.733]	-	0.741
Professionals	71	0.527	[−0.027,1.082]	-	0.836
Translation Directions	0.118				
L2–L1	154	0.493	[0.279,0.707]	-	0.732
L1–L2	47	0.328	[−0.020, 0.676]	-	0.339
Language Family	1.237				
Same Language Family	71	0.353	[0.076, 0.630]	-	0.268
Different Language Family	122	0.554	[0.329, 0.779]	-	0.268
Post-editing Attitudes	4.124				
Reported Post-editing Attitudes	12	−0.031	[−0.553, 0.491]	-	0.044
Non-reported Post-editing Attitudes	181	0.534	[0.363, 0.705]	-	0.044
Machine Translation Systems	0.002				
Google	136	0.470	[0.259, 0.680]	-	0.964
Non-Google	50	0.603	[0.321, 0.884]	-	0.213
Error Types	1.130				
Reported Error Types	48	0.649	[0.280, 1.017]	-	0.289
Non-reported Error Types	145	0.423	[0.225, 0.622]	-	0.289
Text Types	0.822				
Informative Texts	128	0.400	[0.190, 0.611]	-	0.000
Expressive Texts	53	0.642	[0.113, 1.172]	-	0.018
Operative Texts	80	0.668	[0.233, 1.103]	-	0.003
Measurement Tools	0.151				
Eyelink	55	0.459	[0.092, 0.825]	-	0.014
Gazepoint	25	0.574	[−0.003, 1.152]	-	0.051
Tobii	61	0.509	[0.196, 0.823]	-	0.002
Others	52	0.372	[−0.009, 0.752]	-	0.056
Cognitive Types	0.395				
Cognitive Effort	128	0.551	[0.314, 0.788]	-	<0.001
Cognitive Demand	18	0.303	[−0.525, 1.132]	-	0.471
Cognitive Memory	6	0.130	[−0.736, 0.995]	-	0.768
Cognitive Load	30	0.344	[−0.087, 0.774]	-	0.117
Cognitive Attention	11	0.398	[−0.403, 1.199]	-	0.328

Note: Omnibus F-values test whether effect sizes differ across levels of each moderator (i.e., moderator effects). Level-specific *p*-values indicate whether the effect size within each category differs significantly from zero (intercept tests) and should not be interpreted as evidence that the moderator explains between-study heterogeneity.

## Data Availability

The datasets generated during the study are stored on Mendeley Data (doi: 10.17632/vtnnm68zr5.1).
